# Natural diversity of CRISPR spacers of *Thermus*: evidence of local spacer acquisition and global spacer exchange

**DOI:** 10.1098/rstb.2018.0092

**Published:** 2019-03-25

**Authors:** Anna Lopatina, Sofia Medvedeva, Daria Artamonova, Matvey Kolesnik, Vasily Sitnik, Yaroslav Ispolatov, Konstantin Severinov

**Affiliations:** 1 Institute of Molecular Genetics, Russian Academy of Sciences, Moscow, Russia; 2 Institute of Gene Biology, Russian Academy of Sciences, Moscow, Russia; 3 Skolkovo Institute of Science and Technology, Skolkovo, Russia; 4 Pasteur Institute, Paris, France; 5 Department of Physics, University of Santiago de Chile, Santiago, Chile; 6 Waksman Institute, Department of Molecular Biology and Biochemistry, Rutgers, The State University of New Jersey, Piscataway, NJ, USA; 7 Department of Molecular Genetics, Weizmann Institute of Science, Rehovot 76100, Israel

**Keywords:** CRISPR, *Thermus*, diversity of spacers, *Thermus* phages

## Abstract

We investigated the diversity of CRISPR spacers of *Thermus* communities from two locations in Italy, two in Chile and one location in Russia. Among the five sampling sites, a total of more than 7200 unique spacers belonging to different CRISPR-Cas systems types and subtypes were identified. Most of these spacers are not found in CRISPR arrays of sequenced *Thermus* strains. Comparison of spacer sets revealed that samples within the same area (separated by few to hundreds of metres) have similar spacer sets, which appear to be largely stable at least over the course of several years. While at further distances (hundreds of kilometres and more) the similarity of spacer sets is decreased, there are still multiple common spacers in *Thermus* communities from different continents. The common spacers can be reconstructed in identical or similar CRISPR arrays, excluding their independent appearance and suggesting an extensive migration of thermophilic bacteria over long distances. Several new *Thermus* phages were isolated in the sampling sites. Mapping of spacers to bacteriophage sequences revealed examples of local acquisition of spacers from some phages and distinct patterns of targeting of phage genomes by different CRISPR-Cas systems.

This article is part of a discussion meeting issue ‘The ecology and evolution of prokaryotic CRISPR-Cas adaptive immune systems’.

## 1. Introduction

Bacteriophages are the most abundant and ubiquitous biological entities on the planet [1,2]. Viruses of bacteria have profound influence on population and community structure and microbial evolution [3]. Being constantly under viral predation, bacteria have developed a broad range of mechanisms against phages such as CRISPR-Cas systems, restriction–modification systems, abortive infection systems as well as dozens of others, which are yet poorly investigated [4–6]. CRISPR-Cas systems comprise CRISPR DNA arrays with identical repeats and variable spacers, and CRISPR-associated (*cas*) genes [7]. At one end of the CRISPR array, a leader sequence containing a promoter from which the array is transcribed is located [8]. New spacers can be acquired from the genomes of viruses or plasmids. The spacer is acquired at the leader-proximal end of the array and the acquisition of a spacer also leads to the appearance of an additional copy of the CRISPR repeat. Thus, spacers that are located distal to the leader have been acquired earlier than leader-proximal spacers. The CRISPR array is transcribed and the resulting precursor RNA is processed into individual CRISPR RNAs (crRNAs) each containing a spacer sequence and fragments of flanking repeats [8,9]. Individual crRNAs are bound by Cas effector proteins and can recognize nucleic acids complementary to the crRNA spacer. Upon recognition, foreign nucleic acids are destroyed. In DNA targeting CRISPR-Cas systems, spacers in the CRISPR array are not recognized as, in addition to complementarity with the crRNA spacer, the target must also have a protospacer adjacent motif (PAM) recognized by the effector. Since the part of the CRISPR repeat that is located in the place of PAM is not recognized, discrimination of self from non-self becomes possible. Currently, CRISPR-Cas systems are divided into two classes, six types and 33 subtypes that differ in Cas effector components, details of target recognition, target destruction and self versus non-self discrimination [10].

Analysis of CRISPR spacers is a valuable source of information about virus–host interactions, because short DNA fragments of previously encountered viruses are ‘recorded’ in CRISPR arrays as spacers, and cells carrying protective spacers are expected to gain an advantage and become more numerous. Such analysis can be particularly powerful when applied to metagenomic data. Besides extraction from metagenomic data or CRISPR loci [11,12], CRISPR spacers can be directly amplified and analysed either from individual bacterial isolates or from whole communities [13–15].

Comparison of CRISPR arrays from isolated populations of the same species revealed great diversity of spacer sequences, which is increased towards the leader-proximal end of arrays [12,16–18]. Analysis of changes of spacer content over time provided examples of new spacers acquisition to the leader-proximal ends of CRISPR arrays, deletion of old spacers from leader-distal ends and recombination of CRISPR arrays between different strains [14,19–21].

CRISPR spacers can be used to identify viral sequences in metagenomes and monitor changes in viral populations [11,22,23]. Examples of spacers that preferably target local phages from the same sampling site were reported [19,24–26]. Theoretical models of coevolution of viruses and hosts demonstrated the efficiency of CRISPR-Cas defence when viral density is small [27]. Host and virus populations were predicted to oscillate short term, with a few dominant strains existing at every given time point [28]. The presence of multiple spacers against a viral genome in host strains makes it more difficult for virus to escape by acquiring mutations in the targeted sites. This may help to maintain spacer diversity over longer time scales [29].

In this work, we investigated the diversity of CRISPR spacers of uncultured communities of *Thermus* strains from distant hot springs and compared them with each other and with a *Thermus* CRISPR database. We also compared *Thermus* bacteriophages and spacers obtained from the same locations. Our analysis reveals, on the one hand, evidence of CRISPR spacer acquisition by *Thermus* communities from local phages and, on the other hand, global distribution of many spacers and arrays suggesting intercontinental migration of at least some *Thermus* strains between their unique ecological niches.

## 2. Material and methods

### (a) Sample collection

The samples were collected from hot gravel of Mount Vesuvius (October 2014, October 2018) or hot springs at Mount Etna (October 2012), the el Tatio region of northern Chile (October 2014), and the Termas del Flaco region of southern Chile (December 2013 and March 2016) and Uzon caldera in Kamchatka, Russia (August 2018). During collection, samples of gravel were taken 5–100 m from each other and water samples were collected from separate hot springs located within a similar distance. In the case of Termas del Flaco, the same hot springs were sampled in 2013 (two samples) and 2016 (three samples). The samples were stored at 4°C and brought to the laboratory within one to two weeks after collection for analysis. Preliminary experiments with laboratory *Thermus thermophilus* strains HB8 and HB27 revealed no loss of viability during conditions and times of storage used. Vesuvius 2018 samples were analysed 2 days after collection.

### (b) Enrichment cultures

Five millilitres of TB medium [0.8% (w/v) tryptone, 0.4% (w/v) yeast extract, 0.3% (w/v) NaCl, 0.5 mM MgCl_2_ and 0.5 mM CaCl_2_] were inoculated with a 100 µl aliquot of hot spring water sample and incubated overnight at 70°C with vigorous agitation. Enrichment cultures were checked for the presence of *Thermus* by PCR with oligonucleotide primers specific for *Thermus* 16S rRNA gene (electronic supplementary material, table S1). Amplifications were carried out with Taq DNA polymerase under the following conditions: initial denaturation for 5 min at 95°C, followed by 28 cycles of 30 s at 95°C, 30 s at 55°C and 40 s at 72°C, and a final extension at 72°C for an additional 2 min.

### (c) Phage isolation

*Thermus thermophilus* strains HB8 ATCC 27634 and HB27 ATCC BAA-163 were used in enrichment cultures to isolate bacteriophages from environmental samples. Five millilitres of TB medium were inoculated with a 100 µl aliquot of overnight culture of one of the *Thermus* strains and growth proceeded until OD600 reached approximately 0.4. An amount of 0.2–0.5 ml of environmental sample was added and incubation was continued overnight at 70°C with vigorous agitation. To isolate individual phage plaques, 1 ml of enrichment culture was centrifuged for 15 min, and 100 µl aliquots of supernatant were combined with 150 µl of freshly grown *T. thermophilus* HB8 or HB27 cultures (OD600 approx. 0.4). Melted soft (0.75%) TB agar was added, mixtures were poured over 2.5% TB agar plates and incubated overnight at 65°C. Individual plaques were picked with toothpicks and cleaned by several passages on the host *Thermus* strain as described above.

### (d) Phage DNA extraction and sequencing

Phage lysates were prepared and DNA was extracted as described previously [30]. Five hundred nanograms of phage DNA were used for library preparation and pair-end sequencing was carried out on the Illumina MiSeq platform with MiSeq reagent kit v. 2 (Illumina, USA) as described previously [31].

### (e) Phage genome annotation

Phage genomes were automatically annotated using GeneMark [32] and annotation was further manually checked by the Artemis program [33] and verified by Blastp and HHpred programs. The BlastN tool was used to compare the genomes of newly isolated phages with the database.

### (f) Bacterial DNA extraction, amplification and sequencing

DNA was extracted from 2 ml of spring water, mud samples from gravel or enrichment cultures using Blood and Tissue kit (Qiagen) according to the manufacturer's protocol for Gram-negative cells. Different sets of oligonucleotide primers were used to amplify CRISPR spacers (electronic supplementary material, table S1). Amplification was carried out with Taq DNA polymerase under the following conditions: initial denaturation for 5 min at 95°C, followed by 28 cycles of 30 s at 95°C, 30 s at 50–60°C and 40 s at 72°C, and a final extension at 72°C for an additional 2 min. Two nanograms of total DNA were used as a template for each PCR reaction. To avoid biases during PCR amplification, 10 replicates of each PCR reaction were performed for every sample and mixed before further manipulations. Amplicons were visualized on 1% ethidium bromide-stained agarose gel and DNA fragments of 200–1000 bp in length were purified from the gel and sequenced on the Illumina MiSeq platform as described above.

### (g) Spacer clustering and analysis

Raw reads were demultiplexed, trimmed by quality with Phred score greater than or equal to 20 and no admission of ambiguous bases using CLC Genomics 8.0 workbench software (CLC Bio Aarhus, Denmark). Spacers were extracted using spget (https://github.com/zzaheridor/spget). To decrease the number of spacers and to avoid overrepresented diversity because of mistakes during PCR and sequencing, spacers were clustered using UCLUST algorithm [34]*.* The maximum number of substitutions allowed for spacers within one cluster corresponds to 85% identity over the full length of the spacer; end gaps were allowed with zero penalties. Chao index, α and β diversities were calculated with vegan package for R [35]. Good's criterion is defined as 1−(*n*1/*N*), where *N* is a total number of spacers in the sample, and *n*1 is a number of singleton spacers.

Centres of spacer clusters (the most highly represented sequence within a cluster) were compared against the NCBI nucleotide collection (nt) and a local database of *Thermus* phages and plasmids with the BLASTn algorithm with parameters for short input sequences (word size 8). Sequences with more than 85% of identity over the entire spacer length and without indels were considered as positive hits.

PAM identification was performed using the CRISPRTarget online tool [36]. Eight nucleotides upstream and downstream of each protospacer were extracted and used for PAM logo search with the Weblogo online tool (http://weblogo.berkeley.edu/logo.cgi). Repeats sequences from identified CRISPR arrays were classified using the CRISPRmap tool [37].

### (h) Data access

Phages sequences of phiFa, phiKo, phiLo, phiMa and YS40-Isch were deposited in GenBank under accession numbers MH673671, MH673672, MH673673, MH673674 and MK257744, respectively. Sequences of CRISPR spacers from natural *Thermus* communities are available in the electronic supplementary material.

## 3. Results

### (a) The diversity of CRISPR spacers in complete *Thermus* genomes

Fully sequenced genomes of 26 *Thermus* strains isolated around the world were available in GenBank at the time of writing (electronic supplementary material, table S2). *Thermus* genomes usually contain multiple CRISPR-Cas systems of different types [38] located on the chromosome and/or on megaplasmids present in some isolates. Most *Thermus cas* operons have an adjacent CRISPR array with a specific repeat sequence. Because of a clear connection between the type of *Thermus cas* operon and repeat sequence of adjacent CRISPR array [39], each array (and repeat) can be assigned to a specific CRISPR type or subtype. The III-A and III-B subtype *cas* gene operons have adjacent CRISPR arrays with identical repeat sequences. Moreover, it has been shown that effectors of both subtypes III-A and III-B bind to common crRNAs [40,41]. Therefore, the III-A and III-B subtypes cannot be distinguished from each other and are treated here as a single type, type IIIAB.

**Figure 1.**
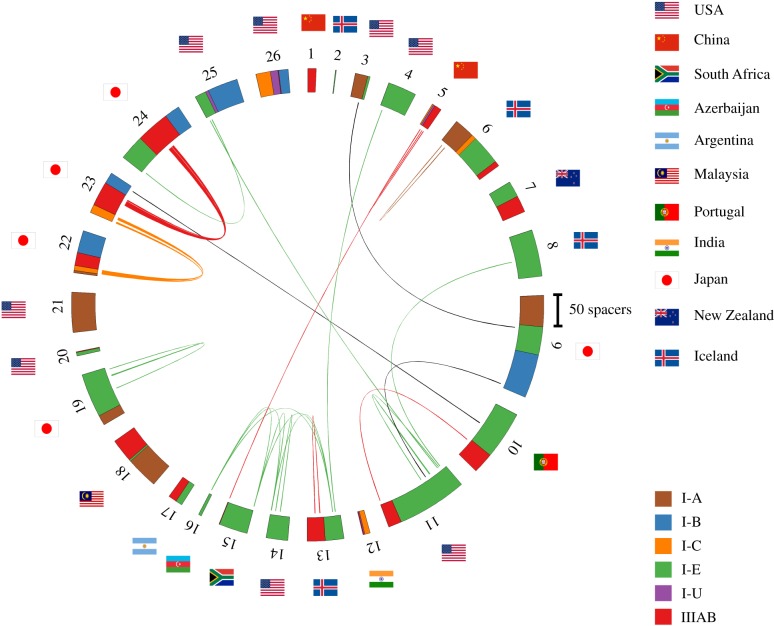
The diversity of CRISPR spacers in fully sequenced *Thermus* genomes. A total of 1567 spacers present in 26 fully sequenced *Thermus* sp. genomes are shown on a circular diagram. *Thermus* isolates used for analysis are numbered outside the spacer diagram (a full list of isolates can be found in electronic supplementary material, table S2). Spacers belonging to arrays of the same CRISPR-Cas systems types/subtype are indicated by identical colours. Spacers that differ from each other by fewer than two nucleotides are connected by lines whose colours correspond to colours indicating CRISPR-Cas systems types/subtypes. Spacers shared by arrays of different types/subtypes are connected by black lines. National flags indicate countries where each strain was isolated.

For further analysis, we considered six dominant *Thermus* CRISPR-Cas systems: I-A, I-B, I-C, I-E, I-U and IIIAB. Consensus repeat sequences for each system used in our analysis are listed in table 1. We used the *spget* program to extract spacers associated with each consensus repeat sequence from fully sequenced *Thermus* genomes and analysed their diversity. Spacers from different *Thermus* isolates were considered identical if they had fewer than two mismatches in their sequences. In this way, a set of 1567 unique *Thermus* spacers was obtained. Most spacers were found to be strain-specific. For very closely related *T. thermophilus* strains isolated in Japan (labelled as 22, 23 and 24 in figure 1), 19 out of 269 spacers were identical and located one after another in CRISPR arrays of the same type. In *T. scotoductus* strains (labelled as 13–16, figure 1), the oldest, leader-distal spacer in one of the I-E CRISPR arrays was shared [42]. Finally, seven pairs of shared spacers must have been independently acquired from the same locus as they were found in CRISPR arrays belonging to CRISPR-Cas systems of different types and/or were partially overlapping. Similar instances of independent spacer acquisition were reported earlier in other microbes [12].

**Table 1.** Types of CRISPR repeats present in *Thermus* sp*.* CRISPR arrays. Consensus sequences built using repeat sequences present in CRISPR arrays of fully sequenced *Thermus* genomes listed in figure 1 are shown.

*N*	type of CRISPR-Cas	repeat sequence	average length of spacer
1	III	GTTGCAMRRGWKKSWKCCCCGYMAGGGGATKRHYDC	41
2	I-E	GTAGTCCCCACRCRYGTGGGGATGGMCSD	32
3	I-C	GTTGCACCGGCCCGAAAGGGCCGGTGAGGATTGAAAC	38
4	I-B	GTTGCAAACCYCGTYAGCCTCGTAGAGGATTGAAAC	36
5	I-U	GTTGCATCCAAGCTTCACAGCTTGGCTACGTTGCAGG	36
6	I-A	GTTTCAAACCCTYATAGGTACGGTYMRAAG	36

In total, only 31 *Thermus* spacers (2.0%) were found in more than one genome (see electronic supplementary material, table S3). For comparison, in a well-studied I-E CRISPR-Cas system of *Escherichia coli,* 90.9% of spacers were shared between at least two isolates (data not shown). These observations imply that the diversity of *Thermus* CRISPR spacers in current databases is very undersampled. BlastN analysis of 1567 unique spacers revealed, respectively, 52 (3.3%) and 80 (5.1%) spacers matching *Thermus* phages and prophages, 14 (0.9%) matches to plasmids and 48 (3.1%) matches to *Thermus* chromosomes in locations other than CRISPR arrays. Most spacers that matched DNA fragments from *Thermus* phages were from I-E and I-B arrays (21 and 20, respectively), suggesting that I-E and I-B systems are most active against known *Thermus* phages.

### (b) Amplification of CRISPR spacers from natural *Thermus* communities

Given that spacer diversity in known *Thermus* genomes is underestimated, we decided to investigate spacer diversity in natural *Thermus* communities by amplifying spacers associated with specific repeats from samples collected from Mount Vesuvius hot gravel, and hot springs at Mount Etna, the el Tatio region in northern Chile, the Termas del Flaco region in southern Chile and Uzon caldera in Kamchatka, Russian Far East. At each collection site, the temperature was within 65–70°C and the pH was neutral, so we expected to find *Thermus* there. Degenerate partially self-complementary primers corresponding to each of the six *Thermus* CRISPR repeat consensus sequences (electronic supplementary material, table S1) were used for PCR amplification. The procedure (electronic supplementary material, figure S1A) was previously used to characterize spacer diversity in various environmental samples [31,43]. As the procedure was not previously applied to *Thermus* CRISPR arrays, primer pairs for I-B, I-E and IIIAB repeats were validated using DNA purified from *T. thermophilus* HB8 strain which harbours the corresponding CRISPR-Cas systems. With each primer pair, a characteristic ladder of amplification products was observed (an example of PCR fragments obtained with primers specific for the I-E type repeat is shown in electronic supplementary material, figure S1B, lane 3). We did not observe amplification products when DNA prepared directly from environmental samples was used as a template for PCR (as an example, see electronic supplementary material, figure S1B, lanes 4 and 5), probably because of the low concentration of *Thermus* cells. However, robust amplification products were seen with DNA prepared from enrichment cultures grown overnight at 70°C in rich medium (see Material and methods). The observed amplification patterns were reproducibly distinct for enrichment cultures seeded with material from different locations (as an example, see electronic supplementary material, figure S1B, lanes 6 and 7).

### (c) The diversity of CRISPR spacers in *Thermus* communities

For each site, amplified material corresponding to spacers from different arrays was combined and subjected to Illumina sequencing. Using a spacer extraction pipeline similar to the one described earlier [31,43], a total of approximately 17.8 million spacers (defined as sequences of an expected length located between two repeats sequences of the same type) were extracted. Spacers with identity of more than 85% over their entire length and belonging to the CRISPR arrays with repeats of the same type were clustered, separately for each sample. The most abundant sequence in a cluster was considered as the cluster centre. Overall, implementation of the procedure described above resulted in 109 843 clusters. We measured α-diversity (Shannon entropy) for each sample and calculated the coverage of spacer diversity based on the number of lowly abundant clusters (see electronic supplementary material, table S4). The lowest coverage was observed for Vesuvius samples 3–6 (27–31%) and Uzon samples 3–5 (25–35%). Given undersequencing of spacers with low abundance, further analysis was performed for clusters that contained more than 10 spacers (14 872 clusters). For simplicity, below we will refer to cluster centres as ‘spacers’.

When spacers from different sites were compared, 7246 unique spacers were identified. The collection of *Thermus* spacers obtained from environmental samples exceeds the number of spacers from sequenced isolates by more than fourfold (7246 compared with 1567). Yet, only 1.2% of spacers from natural *Thermus* communities are similar to database spacers. This value becomes even smaller if minor, less abundant spacers revealed by our analysis, are considered. The result emphasizes the extent of diversity of CRISPR spacers in *Thermus* and, presumably, reflects the high level of activity of *Thermus* CRISPR-Cas systems in spacer acquisition.

**Figure 2.**
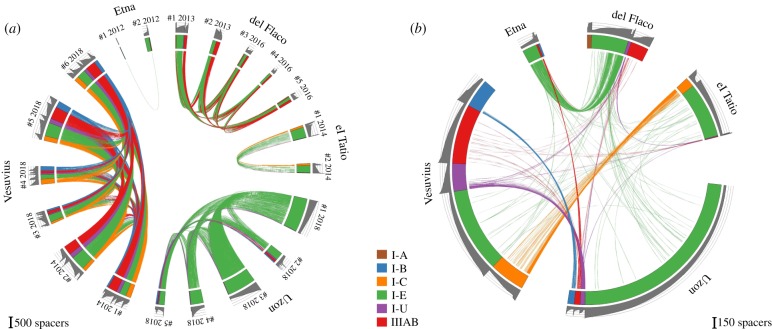
The diversity of CRISPR spacers in environmental *Thermus* samples. (*a*) The diversity of 14 872 spacers (spacer cluster centres) associated with *Thermus* CRISPR repeats from enrichment cultures obtained from samples collected at indicated sites is shown in the circular diagram. Spacers from the same location that differ from each other by fewer than two nucleotides are connected by matching colour lines. For del Flaco, samples #1 and #2 were collected in December 2013 and samples #3–#5 in March 2016. For Vesuvius, samples #1 and #2 were collected in October 2014 and samples #3–#6 in October 2018. (*b*) Spacers from the same location are merged. The resulting diversity of unique 7877 spacers is shown in the circular diagram. Spacers from different locations that differ from each other by fewer than two nucleotides are connected by matching colour lines. The colour labelling scheme is the same as in figure 1. Grey colour histograms on the outside show cluster size in log_10_ scale.

As the overall number of unique spacers (7246) is considerably less than the sum of spacers present in each site (14 872), it follows that some spacers are present in more than one sample. Spacers shared between samples collected from the same locality/reservoir are shown in figure 2*a*. The number of shared spacers ranges from 1.0% in Etna (because of the low number of spacers in Etna 1 sample) to 66% between Vesuvius 1 and Vesuvius 2 samples. Samples from Termas del Flaco, which were taken 27 months apart, illustrate the temporal stability of spacer content in time, with 37–49% of spacers shared between all samples. Even more dramatically, 36–63% of spacers collected 4 years apart at Vesuvius were also common. Interestingly, the frequencies of occurrence of common spacers (as evidenced by the size of the clusters that contain them) were comparable in samples collected at the same site (0.55–0.98 Pearson's coefficient). It can be argued that the enrichment procedure used to prepare cultures suitable for spacer amplification could have introduced a bias in observed spacer content. The stability of spacer sets in samples collected at the same location but separated by extended periods of time makes this possibility unlikely.

By identifying overlapping spacer pairs and triplets in longer Illumina reads, we reconstructed fragments of *Thermus* arrays containing 10–35 spacers (electronic supplementary material, table S5). As an example, three shared I-A CRISPR array fragments from Thermas del Flaco 1 and Thermas del Flaco 5 samples are shown in electronic supplementary material, figure S2. One array remained unchanged over the course of 27 months, another lost three spacers from the leader-distal end and the third was completely renewed except for one pair of spacers. Overall, these observations are consistent with the existence of stable local *Thermus* communities sharing a conserved set of CRISPR spacers but also show evidence of temporal changes due to spacer acquisition and loss.

Analysis of spacers shared between remote sites is shown in figure 2*b*. For simplicity, all spacers present at the same location were combined to create this figure. Electronic supplementary material, figure S3 shows the results when individual samples from the same locations are treated separately. As can be seen, many spacers are shared between different locations. Four hundred and five spacers were shared between two sites, 78 between three sites and four spacers were shared between four sites. Our analysis revealed, rather strikingly, little overlap between spacer sets present in distant localities at the same continent compared with intercontinental spacer sets. For example, there are less common spacers between the El Tatio and Termas del Flaco sets than between the El Tatio and Vesuvius spacers (*p* < 10^−5^, Fisher's exact test). The same result was obtained from hierarchical clustering of samples by pairwise β-diversity (electronic supplementary material, figure S4). The number of shared spacers also did not correlate with geographical distance (electronic supplementary material, figure S5). At present, we are unable to explain this observation. It is possible that certain physico-chemical properties of water that were not recorded during sample collection are responsible. Careful control of ecological parameters of habitat at the collection sites and extension of analysis presented here to other *Thermus* communities around the world may help resolve this issue.

It could be argued that some spacers were acquired independently in different sites. Several identical partially reconstructed arrays were found in different sites. As chances of independent acquisition of identical spacers in the same order are negligible, the results show that some CRISPR arrays (and, presumably, strains that contain them) are shared between distant locations. Shared arrays contained sample-specific spacers, which were acquired at the leader-proximal end of the array (see electronic supplementary material, figure S6 for several examples). The result appears to mirror the situation with another thermophile, an archaeon *Sulfolobus*. In the full genome sequence of *Sulfolobus solfataricus* 98/2 isolated in Italy, 107 out of 189 CRISPR spacers are identical to spacers from *S. solfataricus* P2 isolated in the Yellowstone National Park [44]. Similarly, we found that *S. acidocaldarius* N8 from thermal fields in Japan and *S. acidocaldarius* GG12-C01-09 from Yellowstone share 95% of CRISPR spacers (data not shown).

### (d) The provenance of *Thermus* spacers

We next examined sequences of spacers obtained in this work. Most spacers (94.5%) had no matches to the Genbank nucleotide collection, a situation that is typical for all CRISPR-Cas systems [39]. The remaining spacers matched *Thermus* phages (3.3%), small plasmids (0.4%) or non-CRISPR chromosome/megaplasmid sequences of *Thermus* (1.8%). Alignments of protospacers (sequences matching spacers) and their flanking sequences revealed a putative AAG protospacer-associated motif (PAM) on the 5′-protospacer flank for the I-E system, a GGTN PAM for the I-B system and a TTC PAM for the I-C and I-U systems (electronic supplementary material, figure S3). The AAG PAM has also been reported for the *E. coli* I-E system [43].

More than 100 *Thermus* bacteriophages have been isolated [45–49]. However, only eight complete genomes of *Thermus* phages are available in the Genbank database: IN93, p23-77, YS40, TMA, P23-45, P74-26, phiOH3 and phiOH16. Myoviruses YS40 and TMA, inoviruses phiOH3 and phiOH16, and siphoviruses P23-45 and P74-26 have closely related sequences, respectively. In the course of this work, we have isolated, sequenced and annotated five additional *Thermus* bacteriophages from samples that were used for amplification of spacers. Three phages, phiFa, phiKo and YS40-Isch were isolated from Mount Vesuvius samples, and two (phiLo and phiMa) from el Tatio samples. PhiFa is a siphovirus and most of its genes are homologous to long-tailed phages P23–45 and P74–26 isolated earlier in Kamchatka [50]. PhiKo (11 129 bp, 26 ORFs) belongs to *Tectiviridae* phage family. One PhiKo gene product is homologous to the lysozyme of *Thermus* phage 2119, and three others are homologous to proteins encoded by prophage region of *Thermus* sp. 2.9 isolate. PhiLo (178 531 bp, 165 ORFs) and phiMa (51843 bp, 66 ORFs) are myoviruses. Approximately 10% of phiLo proteins are homologous to proteins encoded by other *Thermus* phages (including YS40, TMA, IN93, P74-26), while 60% of phiMa proteins are most similar to proteins encoded by prophage region of *Thermus* sp. 2.9. YS40-Isch is highly similar to YS40 (87% DNA identity, 85% coverage by BLASTn) and TMA (86% identity, 84% coverage) phages earlier isolated in Japan [46,47].

When the five new *Thermus* phage genomes were taken into account, the percentage of matches of unique spacers with phage sequences increased from 3.3 to 6.3%, indicating that the diversity of *Thermus* phages is greatly undersampled. The results of spacer mapping to known *Thermus* phage genomes are summarized in table 2. The overwhelming majority of spacers that matched phiMa and phiKo genomes came from spacer sets from the same localities (*p* < 10^−15^, Fisher's exact test). Only 17 spacers targeted YS40 isolated from Japan, while 33 spacers from Vesuvius matched YS40_Isch, a local phage. Spacers targeting IN93 were present in spacer sets from every sample. On the basis of the abundance of IN93 targeting spacers in different locations, it appears that, unlike the apparently ‘local’ phages such as phiKo and phiMa, the IN93-like phages are globally spread, possibly because of their ability to lysogenize their hosts.

**Table 2.** BlastN hits of spacers from different sites. The number of BlastN hits for ‘not-unique spacers’, i.e. identical spacers found in different sites, is shown. Only hits with greater than 85% identity over entire spacer length are included. Fisher's exact test was used to test for each virus that the number of protospacers depends on the sample site. The resulting *p*-values are given in the last row.

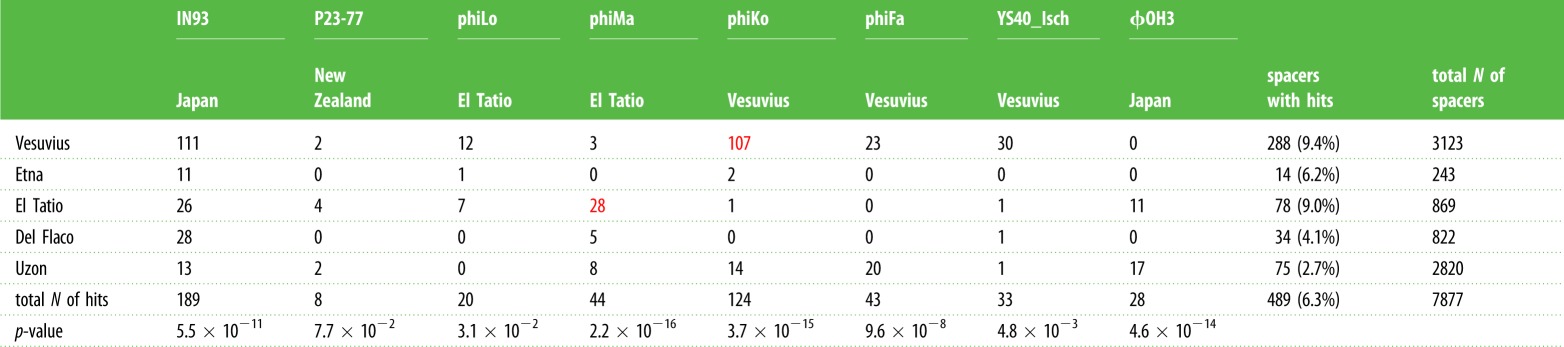

It is apparent that different phages are targeted with widely different frequencies by spacers in our collection. For example, IN93, a small phage with an approximately 20 kb genome, contains 189 protospacers (constituting 38% of the total of phage sequences matching *Thermus* spacers), while some much larger phages, namely YS40-Isch or phiFa, are each targeted by about 30 spacers. It is also apparent that different phages are preferentially targeted by different CRISPR-Cas systems (table 3). Thus, most IN93 targeting spacers belong to the I-E subtype, while phiFa and YS40-Isch are preferentially targeted by IIIAB systems. Interestingly, the I-E system, which contains most unique spacers, has a relatively small percentage of spacers that match phage genomes (4%). This value is significantly higher for spacers of I-C (11%), I-U (9%) and I-B (17%) types.

**Table 3.** BlastN hits of spacers from different CRISPR-Cas systems. The number of hits for ‘not-unique spacers’, i.e. identical spacers belonging to different CRISPR-Cas system types, is shown. Only hits with greater than 85% identity over entire spacer length are included. The predicted PAMs for each system are presented in the second column (PAM logos are shown in electronic supplementary material, figure S3). Fisher's exact test was used to test for each virus that the number of protospacers depends on the type of CRISPR-Cas system. The resulting *p*-values are given in the last row.

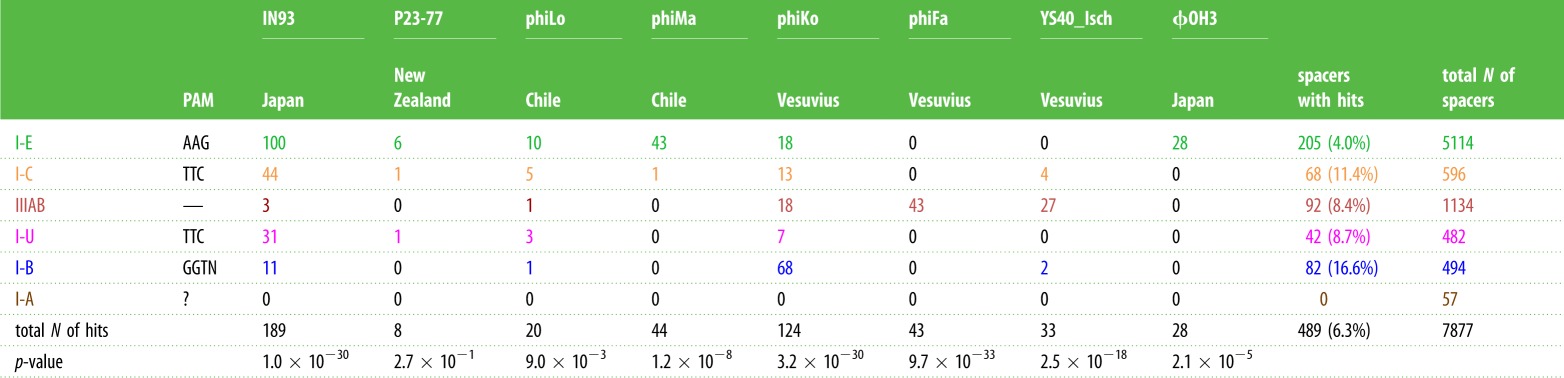

**Figure 3.**
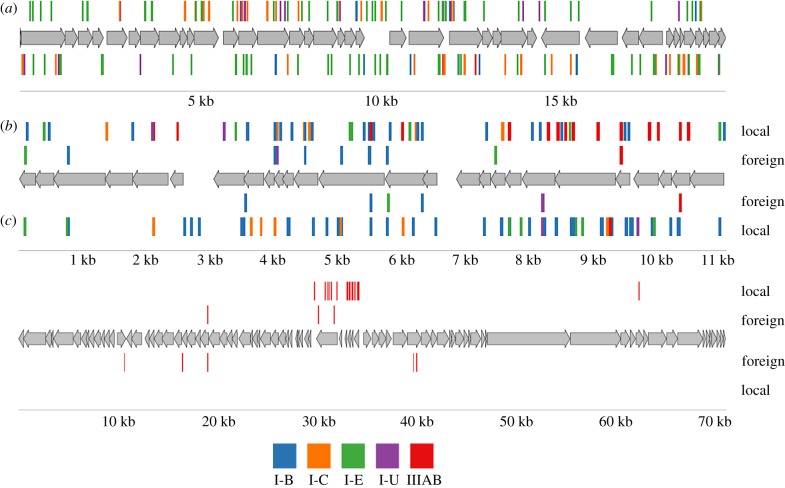
Mapping of protospacers in the genomes of *Thermus* phages. The double-stranded DNA genomes of *Thermus* bacteriophages IN93 (*a*), phiKo (*b*) and phiFa (*c*) are schematically shown. Numbers below indicate genome coordinates, in kilobases. Phage genes are indicated by grey arrows, with arrow directions matching the direction of transcription. Protospacers matching spacers associated with *Thermus* CRISPR repeats are shown as vertical lines above and below phage genomes. The colour of lines representing protospacers indicates the type of CRISPR-Cas systems to which the matching spacers belong (the colour scheme legend is shown at the bottom of the figure). For phiKo and phiFa, mapped spacers are separated into ‘local’, i.e. found at the site of phage isolation, and ‘foreign’, i.e. found at distant sites.

The locations of protospacers in phage genomes that had most matches with *Thermus* spacers—IN93, phiKo and phiFa—are shown in figure 3 and, for phiMa, a phage with a large genome, in electronic supplementary material, figure S2. While the IN93 phage is globally distributed, for phiKo and phiFa phages we performed separate mapping of ‘local’ spacers recovered at the isolation site and ‘foreign’ spacers observed elsewhere. As can be seen from figure 3*b*,*c* and table 2, most spacers matching phiKo and phiFa are local.

The I-E and I-B spacers mapped evenly throughout phage genomes to both DNA strands (figure 3). By contrast, most protospacers matching IIIAB spacers were located on the transcribed strand of viral genes, an expected result given that interference by type III systems is transcription-coupled [51]. The observed location of type III protospacers suggests that phages do exert pressure on *Thermus* communities, for in the absence of such pressure non-functional type III spacers targeting the non-transcribed strand of phage DNA could have been expected. The distribution of type IIIAB protospacers along the genome was also highly uneven in the PhiKo (figure 3*b*) and, most prominently, in the PhiFa genomes (figure 3*c*). In the latter case, protospacers were located in a narrow central region of the genome, where, based on homology to *Thermus* P23-45 phage, the early genes are located. In the case of phiKo, type IIIAB protospacers mapped to the part of the genome where transcription of viral genes likely initiates. The result may indicate that spacers acquired from other regions of phage genomes do not provide bacteria that acquire them protection from the virus and are thus not retained in the population [52]. Alternatively, there may be specific aspects of phage development strategy that limit the adaptation machinery of the host to these regions. The availability of new phages described in this work will allow us to address these questions experimentally.

## Supplementary Material

Supplementary figure S1.

## Supplementary Material

Supplementary figure S2.

## Supplementary Material

Supplementary table S5

## Supplementary Material

Supplementary table S3

## Supplementary Material

Supplementary table S4

## Supplementary Material

Supplementary data 2. Environmental Thermus CRISPR spacers (all)

## Supplementary Material

Supplementary figure S3.

## Supplementary Material

Supplementary data 1. Environmental Thermus CRISPR spacers (majour)

## Supplementary Material

Supplementary table S2

## Supplementary Material

Supplementary figure S4.

## Supplementary Material

Supplementary figure S5.

## Supplementary Material

Supplementary figure S6.

## Supplementary Material

Supplementary figure S7.

## Supplementary Material

Supplementary table S1

## Data accessibility

Sequences of CRISPR spacers from natural *Thermus* communities are available in Supplementary material.
