# Supplementary material for: Natural diversity of CRISPR spacers of Thermus: evidence of local spacer acquisition and global spacer exchange
Source: Philos Trans R Soc Lond B Biol Sci. 2019 Mar 25;374(1772):20180092. doi: 10.1098/rstb.2018.0092 (PMC6452258; doi:10.1098/rstb.2018.0092)
Supplement: Supplementary figure S1. [file rstb20180092supp1.pdf]

Supplementary figure S1. Amplification of Thermus CRISPR spacers from enrichment cultures.

A.

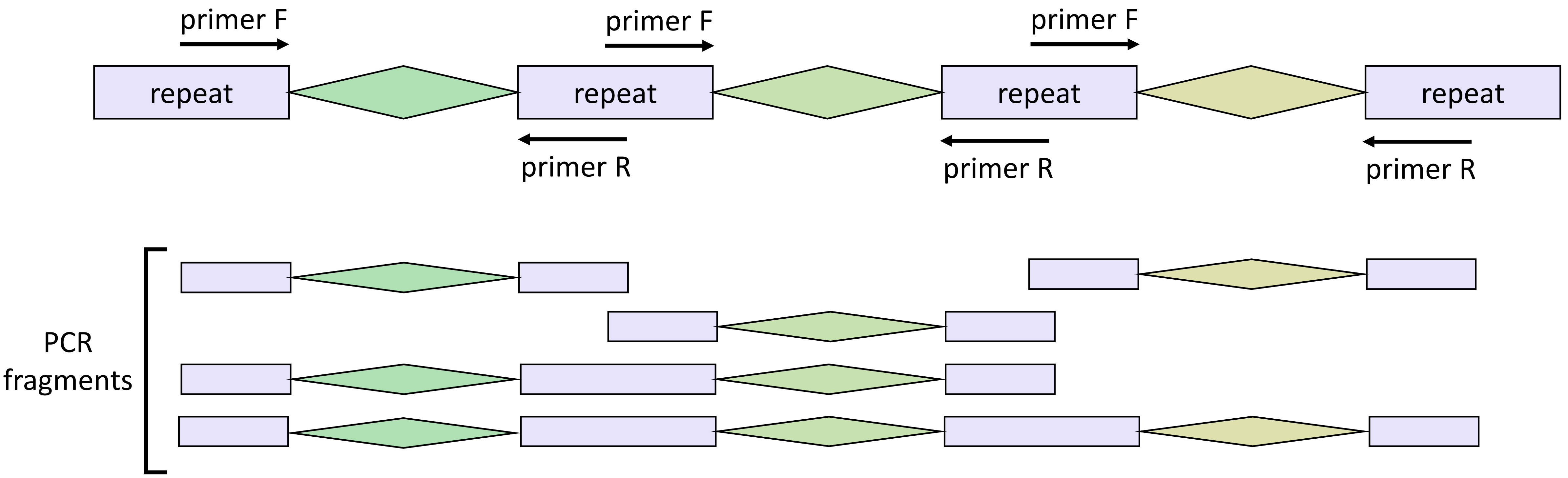

B.

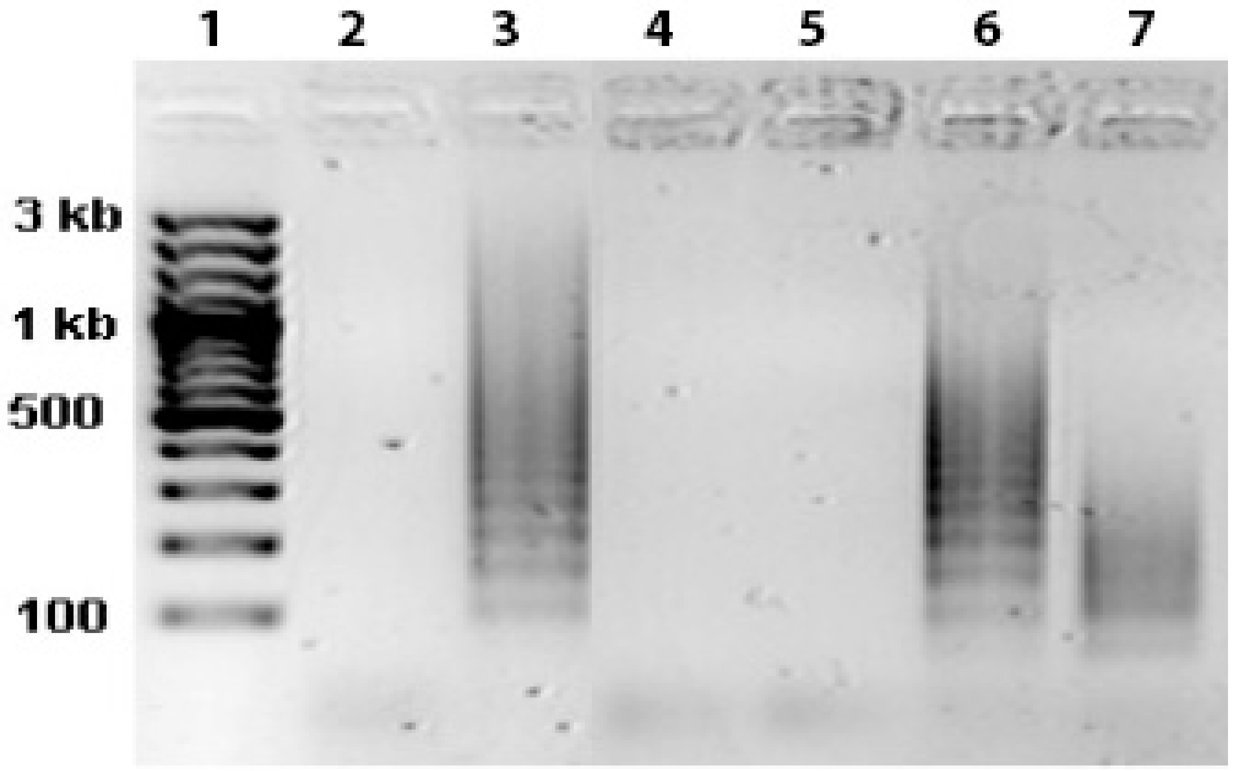

A. A strategy used to amplify spacers associated with Thermus CRISPR repeats from environmental samples.
B. An agarose gel showing the results of separation of products of PCR amplification with primers specific to Thermus I-E type CRISPR repeat using the following DNA as amplification template.
lane 2 - negative control, no input DNA;
lane 3 - T. thermophilus HB8 genomic DNA;
lane 4 - Thermas del Flaco sample 1, no enrichment;
lane 5 - Thermas del Flaco sample 2, no enrichment;
lane 6 - Thermas del Flaco sample 1, enrichment culture;
lane 7 - Thermas del Flaco sample 2, enrichment culture.
lane 1 is a DNA molecular weight marker.
