# Supplementary material for: Natural diversity of CRISPR spacers of Thermus: evidence of local spacer acquisition and global spacer exchange
Source: Philos Trans R Soc Lond B Biol Sci. 2019 Mar 25;374(1772):20180092. doi: 10.1098/rstb.2018.0092 (PMC6452258; doi:10.1098/rstb.2018.0092)

Supplementry Figure S3. The diversity of CRISPR spacers in environmental *Thermus* samples. **T**he diversity of 14872 spacers (spacer cluster centers) associated with *Thermus* CRISPR repeats from enrichment cultures from samples collected at indicated sites is shown in circular diagram. Spacers from different locations that differ from each other by less than 2 nucleotides are connected by matching color lines.

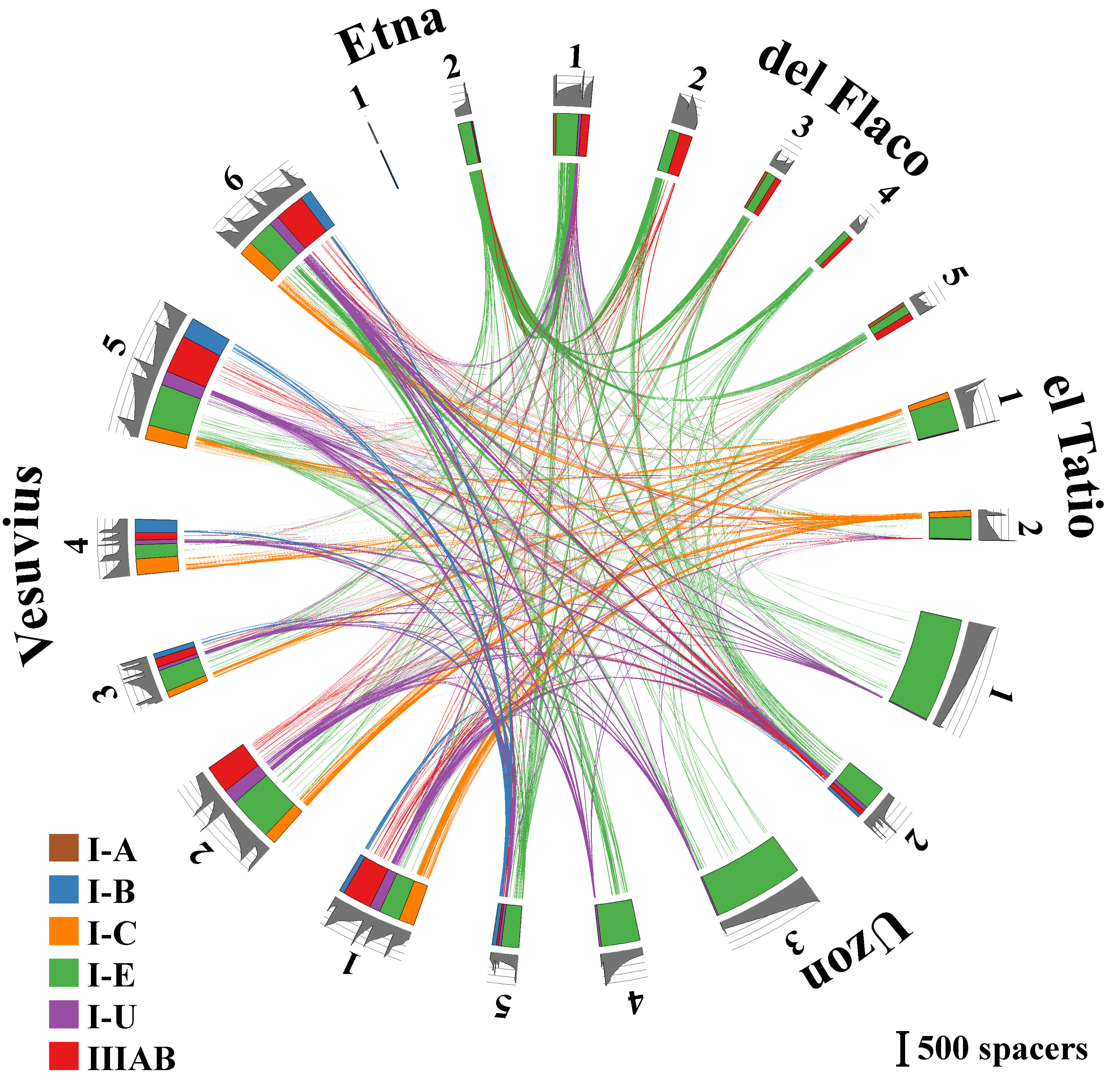

Supplement: Supplementary figure S3. [file rstb20180092supp7.pdf]
