# Supplementary material for: Natural diversity of CRISPR spacers of Thermus: evidence of local spacer acquisition and global spacer exchange
Source: Philos Trans R Soc Lond B Biol Sci. 2019 Mar 25;374(1772):20180092. doi: 10.1098/rstb.2018.0092 (PMC6452258; doi:10.1098/rstb.2018.0092)
Supplement: Supplementary figure S4. [file rstb20180092supp10.pdf]

Supplementary Figure S4. Clustering of samples by pairwise beta-diversity.

A. Grey-scale heatmap with dendrogram of sample clustering
B. Red-scale heatmap with number of shared spacers indicated in each cell

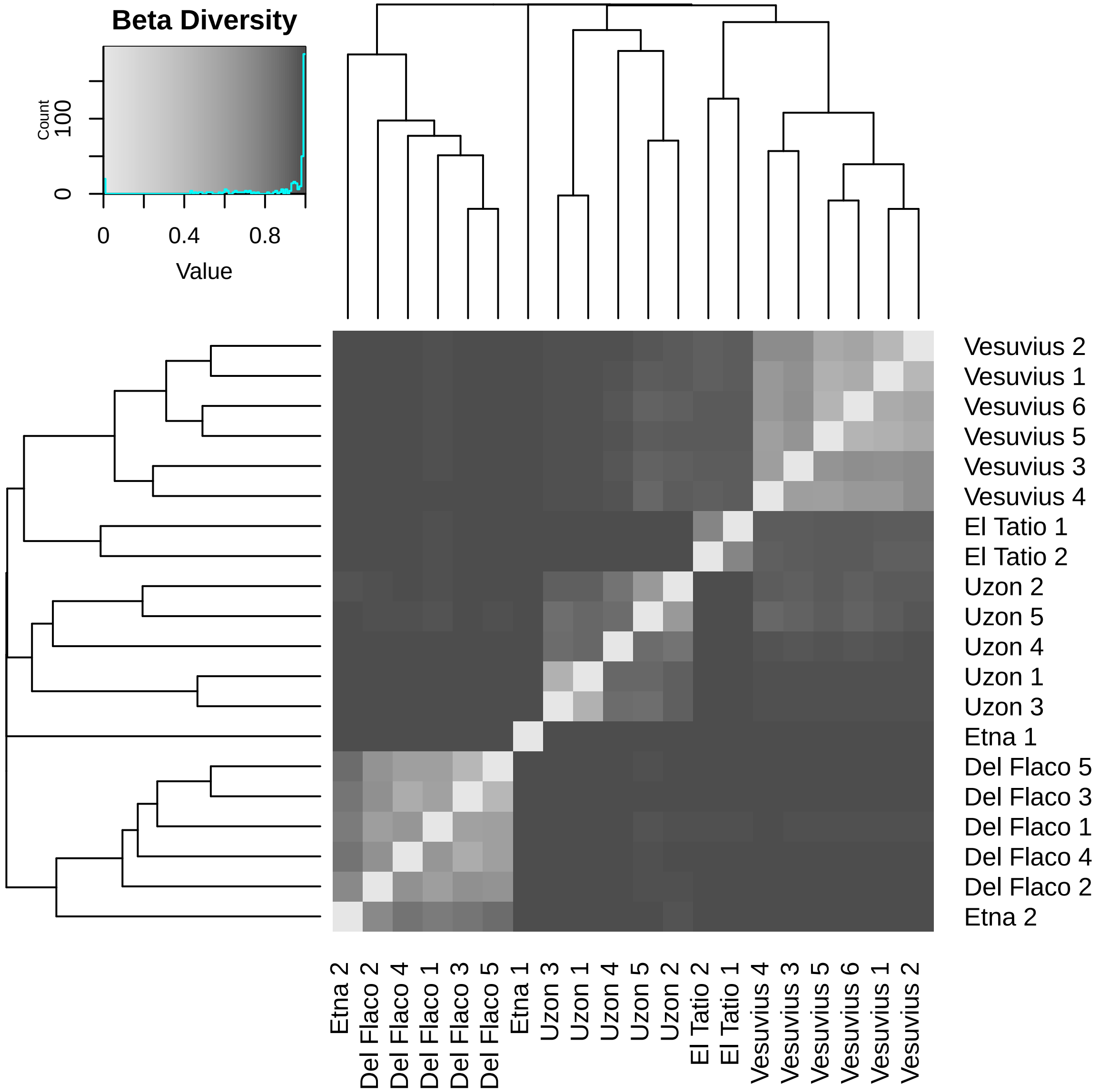

Beta Diversity
Count
0
100
0
0.4
0.8
Value
Vesuvius 2
Vesuvius 1
Vesuvius 6
Vesuvius 5
Vesuvius 3
Vesuvius 4
El Tatio 1
El Tatio 2
Uzon 2
Uzon 5
Uzon 4
Uzon 1
Uzon 3
Etna 1
Del Flaco 5
Del Flaco 3
Del Flaco 1
Del Flaco 4
Del Flaco 2
Etna 2
Etna 2
Del Flaco 2
Del Flaco 4
Del Flaco 1
Del Flaco 3
Del Flaco 5
Etna 1
Uzon 3
Uzon 1
Uzon 4
Uzon 5
Uzon 2
El Tatio 2
El Tatio 1
Vesuvius 4
Vesuvius 3
Vesuvius 5
Vesuvius 6
Vesuvius 1
Vesuvius 2

	Etna 2	Del Flaco 2	Del Flaco 4	Del Flaco 1	Del Flaco 3	Del Flaco 5	Etna 1	Uzon 3	Uzon 1	Uzon 4	Uzon 5	Uzon 2	El Tatio 2	El Tatio 1	Vesuvius 4	Vesuvius 3	Vesuvius 5	Vesuvius 6	Vesuvius 1	Vesuvius 2
Vesuvius 2	4	7	3	19	7	3	1	23	21	26	39	66	79	74	387	366	915	712	853	1432
Vesuvius 1	3	5	1	17	4	1	1	23	21	25	59	58	77	68	425	343	911	721	1187	853
Vesuvius 6	3	3	0	26	2	2	1	30	38	43	87	86	60	57	467	360	1011	1365	721	712
Vesuvius 5	6	9	4	25	7	4	1	28	33	37	82	82	74	69	649	508	1872	1011	911	915
Vesuvius 3	4	4	1	14	3	2	0	25	22	28	54	53	40	45	347	674	508	360	343	366
Vesuvius 4	2	2	0	6	1	2	1	24	23	29	73	52	55	53	812	347	649	467	425	387
El Tatio 1	4	2	3	12	2	2	0	5	3	2	4	2	154	587	53	45	69	57	68	74
El Tatio 2	2	1	2	10	2	1	0	4	4	2	2	2	439	154	55	40	74	60	77	79
Uzon 2	16	12	5	15	5	5	0	78	89	113	205	552	2	2	52	53	82	86	58	66
Uzon 5	3	10	4	15	4	5	0	146	122	74	393	205	2	4	73	54	82	87	59	39
Uzon 4	1	0	0	4	0	0	0	147	144	606	74	113	2	2	29	28	37	43	25	26
Uzon 1	1	0	1	4	1	1	0	888	1559	144	122	89	4	3	23	22	33	38	21	21
Uzon 3	3	0	1	5	0	1	0	1359	888	147	146	78	4	5	24	25	28	30	23	23
Etna 1	1	0	0	0	0	0	18	0	0	0	0	0	0	0	1	0	1	1	1	1
Del Flaco 5	41	125	110	199	182	289	0	1	1	0	5	5	1	2	2	2	4	2	1	3
Del Flaco 3	49	115	126	197	270	182	0	0	1	0	4	5	2	2	1	3	7	2	4	7
Del Flaco 1	91	211	149	539	197	199	0	5	4	4	15	15	10	12	6	14	25	26	17	19
Del Flaco 4	39	99	173	149	126	110	0	1	1	0	4	5	2	3	0	1	4	0	1	3
Del Flaco 2	92	352	99	211	115	125	0	0	0	0	10	12	1	2	2	4	9	3	5	7
Etna 2	226	92	39	91	49	41	1	3	1	1	3	16	2	4	2	4	6	3	3	4
