# Supplementary figures and images for: Natural diversity of CRISPR spacers of Thermus: evidence of local spacer acquisition and global spacer exchange
Source: Philos Trans R Soc Lond B Biol Sci. 2019 Mar 25;374(1772):20180092. doi: 10.1098/rstb.2018.0092 (PMC6452258; doi:10.1098/rstb.2018.0092)

Supplementary Figure S5.

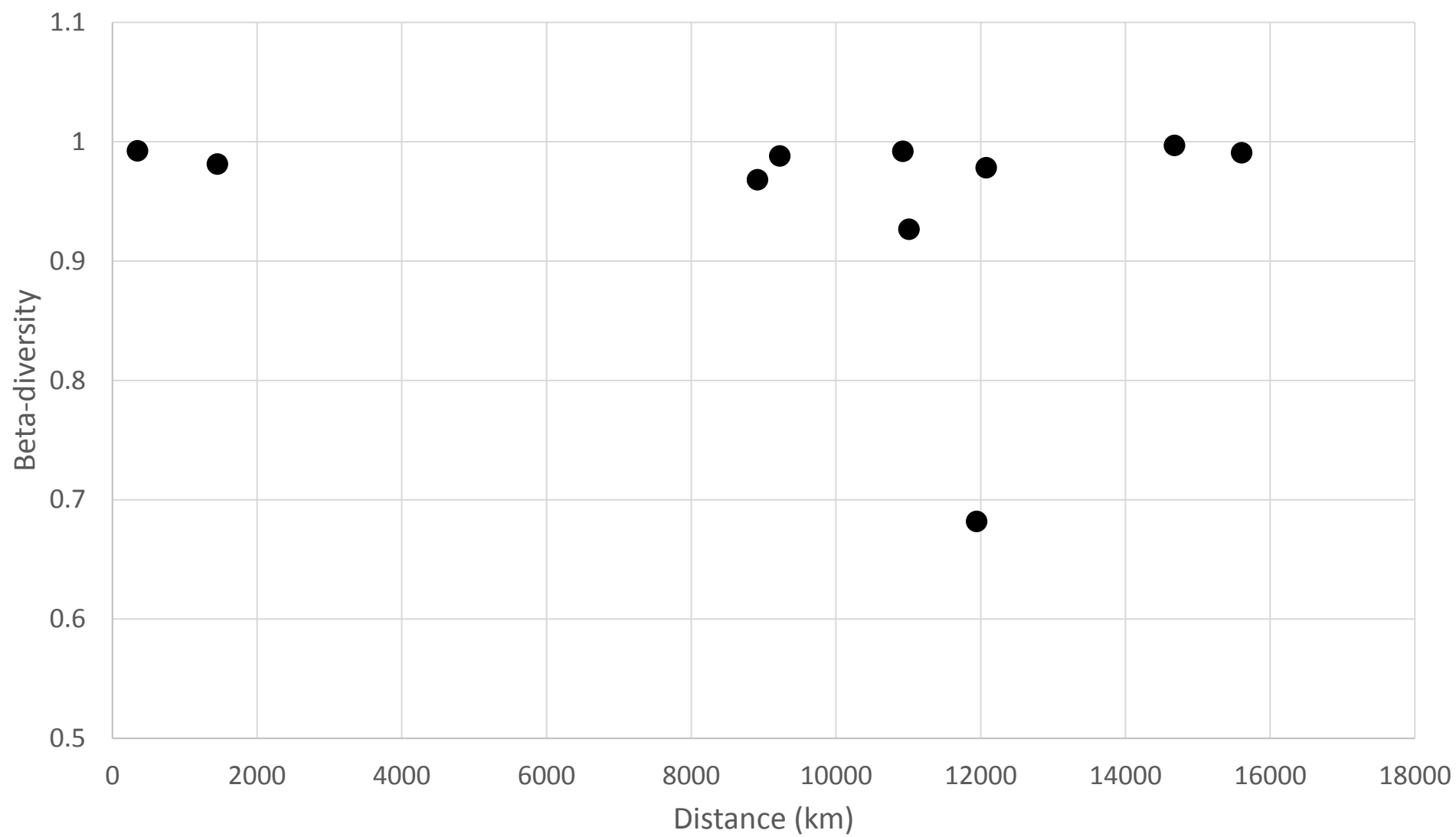

Supplement: Supplementary figure S5. [file rstb20180092supp11.pdf]
