# Supplementary material for: Natural diversity of CRISPR spacers of Thermus: evidence of local spacer acquisition and global spacer exchange
Source: Philos Trans R Soc Lond B Biol Sci. 2019 Mar 25;374(1772):20180092. doi: 10.1098/rstb.2018.0092 (PMC6452258; doi:10.1098/rstb.2018.0092)
Supplement: Supplementary figure S7. [file rstb20180092supp13.pdf]

Supplementary figure S2. PAM motifs identified by matches to *Thermus* phages

I-E system

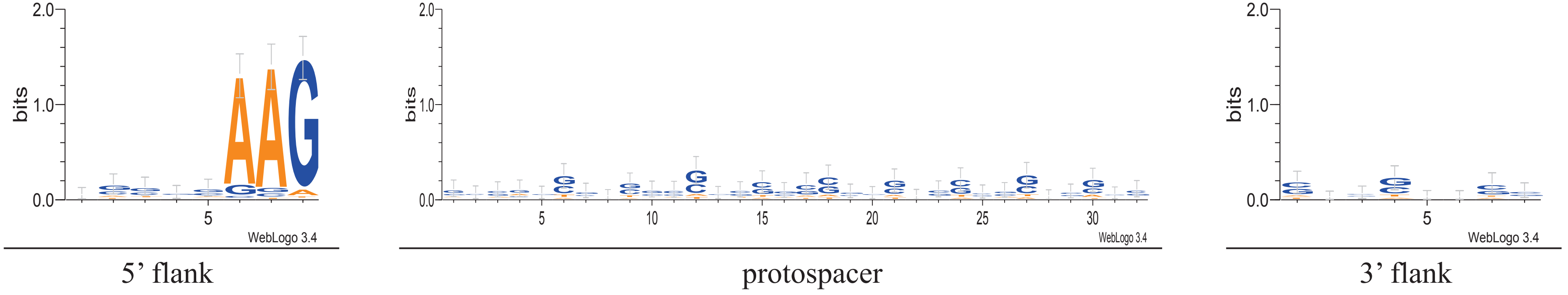

I-B system

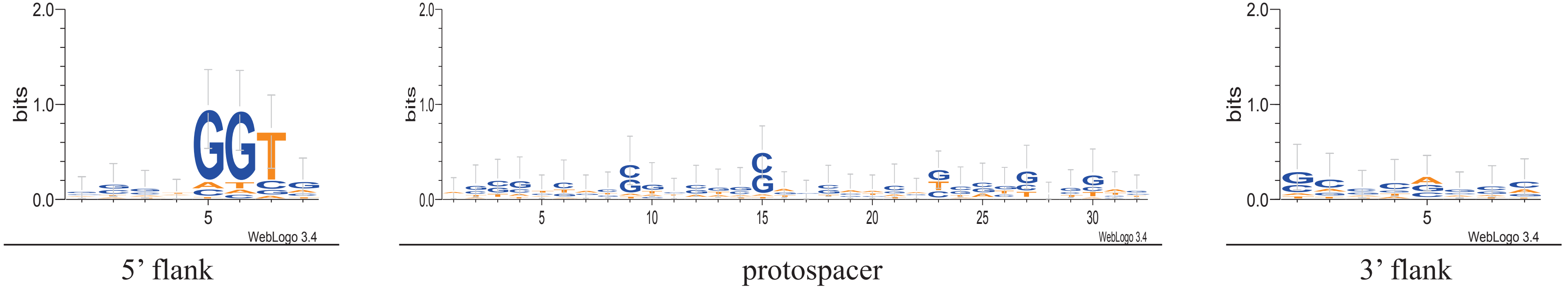

IIIAB system

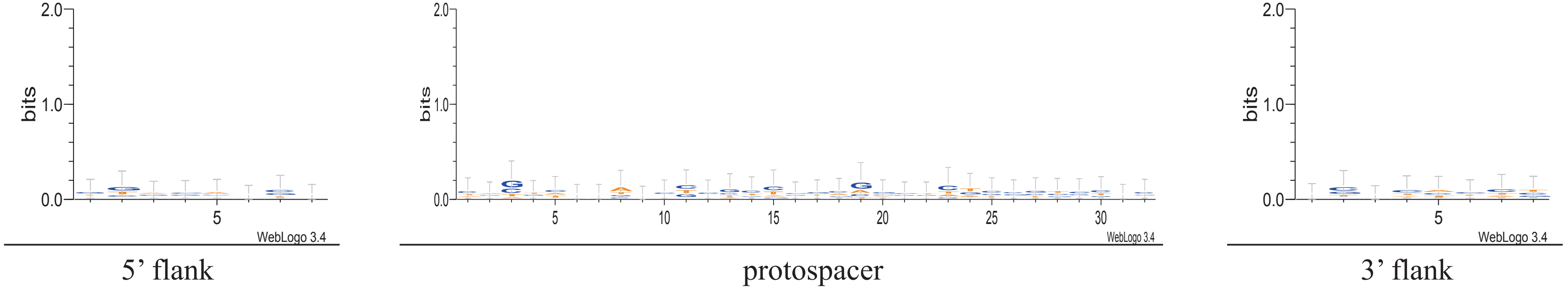

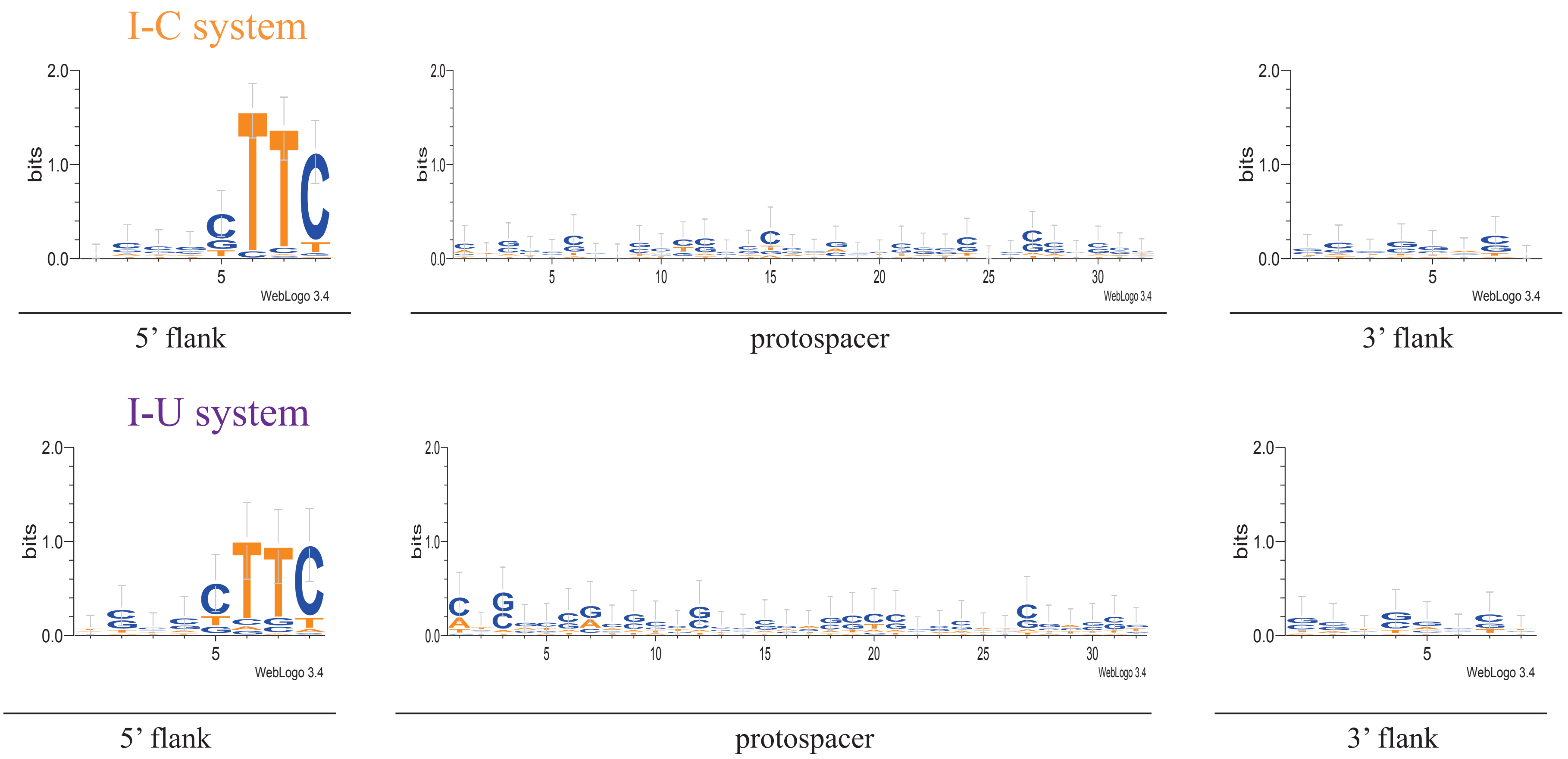

Natural Diversity of CRISPR Spacers of Thermus: Evidence of Local Adaptation and Global Spacer Exchange
